# Role of Morphological and Hemodynamic Factors in Predicting Intracranial Aneurysm Rupture: A Review

**DOI:** 10.7759/cureus.9178

**Published:** 2020-07-14

**Authors:** Srood Jirjees, Zin Mar Htun, Israa Aldawudi, Prakash C Katwal, Safeera Khan

**Affiliations:** 1 Neurology, California Institute of Behavioral Neurosciences & Psychology, Fairfield, USA; 2 Internal Medicine, California Institute of Behavioral Neurosciences & Psychology, Fairfield, USA; 3 Radiology, California Institute of Behavioral Neurosciences & Psychology, Fairfield, USA

**Keywords:** subarachnoid hemorrhage, morphology, and hemodynamic" mesh.", intracerebral aneurysms, risk of rupture

## Abstract

Intracranial aneurysms (IAs) carry the risk of rupture, which will lead to subarachnoid hemorrhage, which has a high mortality and morbidity risk. However, the treatment of IA's carries mortality and morbidity risks too. There are well-known risk factors for the rupture of IAs like age, size, and site. However, choosing patients with unruptured IAs for treatment is still a big challenge. This review article aimed to find out the relationship between morphological and hemodynamic characters of IAs with their rupture and incorporate these factors with well-known factors to yield an accurate module for predicting the rupture of IAs and decision-making in the treatment of unruptured IAs.

We searched in PubMed and Medline databases by using the following keywords: IAs, subarachnoid hemorrhage, and risk of rupture, morphology, and hemodynamic “mesh.” A total of 19 studies with 7269 patients and 9167 IAs, of which 1701 had ruptured, were reviewed thoroughly. Some modules like population, hypertension, age, size, earlier subarachnoid hemorrhage, and site (PHASES) score that involve well-known risk factors can be used to assess the risk of rupture of IAs. However, decision making for treating unruptured IA needs more detailed and more accurate modules. Studying morphological and hemodynamic factors and incorporation of them with well-known risk factors to yield a more comprehensive module will be very helpful in treating unruptured IA. Among morphological factors, aspect ratio (AR), size ratio (SR), aneurysm height, and bottle-neck factor showed significant effects on the growth and rupture of IA. Besides, wall shear stress (WSS), oscillatory shear index (OSI), and low wall shear stress area (LSA) as hemodynamic factors could have a substantial impact on the formation, shape, growth, and rupture of unruptured IA.

## Introduction and background

Intracranial aneurysms (IAs) occur in about 2-3% of general population [1]. Nowadays, the prevalence is even higher, reaching up to 5% due to the wider availability of non-invasive imaging techniques [2]. Most of the IAs are asymptomatic, but if they rupture, the patient will suffer from subarachnoid hemorrhage with high mortality and morbidity rates, which poses a substantial economic burden on the healthcare system [3,4]. However, the treatment of IAs, whether endovascularly or by microsurgery, carries a non-negligible risk of morbidity. Therefore, choosing unruptured IAs for treatment is a big challenge [5,6].

Multiple well-known risk factors are contributing to the formation, growth, and rupture of IAs such as genetics, age, hypertension, smoking, size, and site of the aneurysms [7,8]. However, the actual mechanisms that lead to an aneurysm rupture are not well understood yet. It is known that the hemodynamics of the aneurysm plays a significant role in the pathophysiology of IAs [9]. Recently, computational fluid dynamics has become a popular tool in studying the hemodynamics of the IAs and predicting their rupture risk [10]. It has also been noticed that several morphological parameters may contribute to the IA rupture [11,12]. The morphology and growth of intracranial aneurysm are very complex due to the diverse nature of fluid mechanics. Because of the living nature of blood vessels, mechanical stimuli are transduced into biological signals, triggering inflammatory cascades leading to blood vessel wall remodeling. For this reason, cerebral aneurysmal hemodynamics has a significant role in the aneurysmal biophysical pathogenesis, evolution, and risk of rupture [13].

The treatment decisions of unruptured IAs may improve to a large extent with an accurate prediction model based on the different types of risk factors aiming to identify patients with a high risk of aneurysmal rupture [14]. Treatment of unruptured IAs would be indicated when the risk of rupture from natural history is higher than the risk of treatment and follow-up. Therefore, despite the guidelines, treatment should be individualized, and the expertise of the individual center should be taken into consideration. The risk of treatment at a center depends upon the cerebrovascular expertise of the neurosurgeons and neurointerventionists [15].

This review aims to find out an accurate relationship between hemodynamics and morphology of the aneurysm and the risk of rupture of the IAs in order to select unruptured IAs more accurately for microsurgical or endovascular treatment.

## Review

Methods

We searched thoroughly using PubMed and Medline databases by using the following keywords, both alone and in combination: IA, subarachnoid hemorrhage, and risk of rupture, morphology, and hemodynamic “mesh.” Thirty relevant studies were shortlisted; the duplicate and irrelevant studies were removed after a thorough scan. Inclusion and exclusion criteria were applied. Finally, 11 studies were removed, and a total of 19 studies were included to be reviewed.

Inclusion/Exclusion Criteria

Papers that are relevant to the topic were selected. Research papers published in the English language only and published from 2014 to 2020 were selected for the review. Only full-text articles were selected for the review. The abstracts for which full-text was not retrieved were excluded from the review.

Results

In this review article, we reviewed 19 studies that collectively had 7269 patients and 9167 cases of IAs, of which 1701 had ruptured [13-31]. Fourteen studies were observational studies, three of them were review articles, one was a meta-analysis, and one was a case series. Table 1 summarizes the studies and necessary information regarding the number of patients and aneurysms.

**Table 1:** A summary of the reviewed studies and basic information regarding the number of patients and aneurysms. AWE: aneurysm wall enhancement, PCoA: posterior communicating artery, MCA: middle cerebral artery, WSS: wall shear stress, IA: intracranial aneurysm, AR: aspect ratio, OSI: oscillatory shear index, WSSmin: minimum wall shear stress, LSA: low wall shear stress area, SR: size ratio.

Authors/Year of Publication	Type of the Study	Purpose of the Study	No. of the Patients	No. of the Aneurysms	No. of Ruptured Aneurysms	Result/Conclusion
Lv et al., 2020 [16]	Cohort	To investigate the relationship between morphology, hemodynamics, and AWE on MRI of the vessel wall and their significance in the rupture of IAs.	57	65	65	Unruptured IAs with a higher rupture risk showed a significantly larger size, lower wall shear stress, and more intense AWE.
Detmer et al., 2019 [17]	Cross-sectional	To look for associations of hemodynamics, morphology, patient age, and gender with aneurysm rupture.	1265	1931	N/A	Adverse morphology and hemodynamics are related to rupture as well as younger age, male gender, and bifurcation aneurysms.
Detmer et al., 2019 [18]	Cross-sectional	To develop a model for aneurysm rupture based on hemodynamic and geometric parameters, aneurysm location, and patient gender and age.	1472	2129	616	The model of combined variables discriminated between ruptured and unruptured aneurysms. Internal validation indicated the potential for the application of this model in clinical practice after evaluation with longitudinal data.
Ambekar et al., 2016 [15]	Literature review	To explain the physical and biological interactions that govern aneurysm pathophysiology.	N/A	N/A	N/A	The combined effect of the assessed hemodynamic stressors triggers inflammatory and signaling cascades that ultimately result in vessel wall thinning, dilation, and rupture.
Liu et al., 2019 [19]	Cohort	To explore the hemodynamic-morphological risk factors for the intra-operative aneurysm rupture.	2237	2237	96	AR, NWSSm, and OSI are considered as three independent risk factors for intraoperative aneurysm rupture, which could serve as predictors.
Zhang et al., 2018 [20]	Case-control	To evaluate the impact of morphological and hemodynamic factors on the rupture of matched-pairs of ruptured-unruptured IAs.	40	20	0	Irregular shape, larger size, higher AR, lower WSSmin, and more LSA may indicate a higher risk for their rupture.
Soldozy et al., 2019 [13]	Cross-section	To find out a model for predicting aneurysm rupture based on geometric and hemodynamic parameters, location, age, and gender of the patient.	1061	1631	492	The model was able to distinguish between ruptured and unruptured aneurysms. After internal validation, it was applicable to clinical practice.
Huang et al., 2018 [21]	Cohort	To find out morphometric and hemodynamic analyses in ruptured and unruptured PCoA aneurysms to improve predictive assessment for rupture.	79	79	57	There was the highest incidence of types II + III with the largest surface area, but the lowest incidence of types I + IV with the smallest surface area in ruptured PCoA aneurysms.
Qiu et al., 2017 [22]	Cross-section	To explore the relationship of various morphological characteristics and WSS by measuring morphological indices, WSS of aneurysms, and the parent artery surface.	39	47	N/A	There is an uneven distribution of WSS in the various parts of the pre-aneurysm vessel. AR and SR can affect aneurysm WSS.
Longo et al., 2017 [23]	Literature review	To find the role of hemodynamic forces in the evaluation of the natural history of unruptured IAs.	N/A	N/A	N/A	A competent risk rupture stratification and treatment strategy selection based on hemodynamic forces have not yet been created, and further efforts should be made to accomplish this vital goal.
Song et al., 2017 [24]	Cross-section	To find out the hemodynamic and morphological differences between aneurysmal MCA bifurcation and contralateral nonaneurysmal anatomy.	36	36	24	A larger bifurcation angle was more prevalent in aneurysmal than nonaneurysmal MCA bifurcations and the higher flow resistance caused by the larger bifurcation angle might be a potential hemodynamic factor associated with the MCA aneurysm presence.
Doddasomayajula et al., 2017 [25]	Cross-section	To test whether the differences in hemodynamics between anterior and posterior circulations can explain the difference in rupture rates of IAs.	117	117	36	Large and concentrated inflow jets, complex and oscillatory flow patterns, and WSS distributions in the posterior circulation, especially in the tip of the basilar artery, could explain their increased rupture risk compared with internal carotid bifurcation aneurysms.
Kaneko et al., 2017 [26]	Cross-section	To develop an in vitro model to study the biological effect of complex-flow stress on endothelial cells.	N/A	N/A	N/A	A geometrically realistic IA model with live endothelial lining was successfully developed, which enables studying the biological impact of complex flow on endothelial cells.
Mocco et al., 2017 [27]	Cohort	To use the prospective International Study of Unruptured IAs cohort to identify morphological characteristics predictive of unruptured IA rupture.	255	255	57	Morphological factors, like perpendicular height and SR, may influence unruptured IA rupture risk.
Qiu et al., 2017 [28]	Cohort	To investigate the correlations between intracranial aneurysm morphology and WSS to find out reliable predictors of rupture risk.	63	72	41	Aneurysm morphology might affect the distribution and magnitude of WSS based on differences in blood flow.
Skodvin et al., 2019 [29]	Case series	To compare morphologies from different angiograms obtained before and just after rupture. To evaluate if postrupture morphology can be used to assess the rupture risk.	29	29	29	The changes in aneurysm morphology that were observed after rupture reflect the cumulative effect of time with continuous growth, and formation of irregularities. Postrupture morphology should not be considered in the evaluation of rupture risk.
Detmer et al., 2018 [14]	Review	To discuss the important natural history studies of unruptured IAs and review the existing scientific evidence and recent advances that help identify the rupture risk guide management of Unruptured IAs.	N/A	N/A	N/A	AR, SR, and WSS are the three most promising factors that might have a role in predicting the risk of rupture.
Can and Du, 2015 [30]	Meta-analysis	To quantify the effect of hemodynamic factors on aneurysm formation and their association with the risk of rupture.	499	499	168	An increase in WSS and gradient oscillatory numbers may contribute to aneurysm formation, whereas low WSS is associated with ruptured aneurysms. The location of the aneurysm at the bifurcation or sidewall may affect the correlation of these hemodynamic factors.
Zhang et al., 2014 [31]	Cross-section	To investigate the rupture-related characteristics on 20 matched-pairs of ruptured-unruptured saccular aneurysms located unilaterally on the anterior circulation in the same patient.	20	20	20	The ruptured aneurysms manifested irregular shape, larger size, higher AR, lower WSS minimum, and more LSA compared with their unruptured mates.
Total	___	___	7269	9167	1701	___

Discussion

There are multiple known risk factors of the rupture of an aneurysm such as young age, maximum size ≥ 7mm, female sex, location of the aneurysm (basilar bifurcation, internal carotid-posterior communicating artery, and possibly anterior communicating artery), Finnish and Japanese descent, smoking, hypertension, and history of subarachnoid hemorrhage [14]. Greving et al. developed the population, hypertension, age, size, earlier subarachnoid hemorrhage, and site (PHASES) score to estimate the risk of aneurysm rupture, which corresponds to a five-year risk of rupture [8]. The five-year absolute aneurysm rupture risk increases with the score increasing. Table 2 shows the PHASES score. However, morphological and hemodynamic factors also play a significant role in the formation and rupture of IA.

**Table 2:** PHASES score to estimate the 5-year aneurysm rupture rate [8]. PHASES: population, hypertension, age, size, earlier subarachnoid hemorrhage, and site, ICA: internal carotid artery, MCA: middle cerebral artery, PCoA: posterior communicating artery, SAH: subarachnoid hemorrhage, ACA: anterior cerebral arteries.

PHASES Aneurysm Risk Score	
Criteria	Points
Population	
North American, European (other than Finnish)	0
Japanese	3
Finnish	5
Hypertension	
No	0
Yes	1
Age	
<70 years	0
≥70 years	1
Size of aneurysm	
<7.0 mm	0
7.0-9.9 mm	3
10.0-19.9 mm	6
≥20.0 mm	10
Earlier SAH from another aneurysm	
No	0
Yes	1
Site of aneurysm	
ICA	0
MCA	2
ACA/PcoA/posterior circulation	4

Posterior circulation is composed of two vertebral arteries, basilar artery, cerebellar arteries, and two posterior cerebral arteries. ACA indicates anterior cerebral arteries that include the anterior cerebral artery, the anterior communicating artery, and the pericallosal artery.

The Morphology of Aneurysm

It is believed that morphological factors of IAs have an essential role in the prediction of aneurysm rupture.

Detmer et al. who studied 1931 aneurysms found that aspect ratio (AR) - the ratio of the maximum perpendicular height to the average neck diameter, where the average neck diameter was calculated as twice the average distance from the neck centroid to the edge of the neck - was larger in ruptured aneurysms compared to unruptured ones (p < 0.001) [17]. Liu et al. studied the morphological difference in 96 ruptured and 96 unruptured IAs. They noted that AR in ruptured IAs was 1.8±0.7, whereas it was 1.2±0.5 in unruptured IAs (p < 0.001) [19]. Huang et al. who studied 79 aneurysms discovered that half of the 57 ruptured aneurysms had significantly high AR (p < 0.05) [21]. Qiu et al. who studied 47 aneurysms found that AR was (1.62±0.84) [22]. Doddasomayajula et al. have studied 117 aneurysms of which 36 have ruptured and noticed that ruptured aneurysms had more elongated shapes (depth, p = 0.01; AR, p = 0.001) than unruptured aneurysms [22]. However; another case-control study done by Mocco et al. on 57 ruptured (case) and 197 unruptured (control) aneurysms showed that AR is not a statistically significant predictor of aneurysm rupture [27]. In a case series of 29 ICAs Skodvin et al. discovered that the median AR before rupture was 1.5 (range, 0.8-4.0) compared with 1.9 (range, 0.8-6.7) after rupture (p = 0.008) [29]. Finally, in a cross-section study, Zhang et al. studied 20 ruptured and 20 unruptured IAs and noted that AR is significantly higher in ruptured ICAs (p = 0.004) [31].

The size ratio (SR) of an aneurysm which is the ratio between maximum aneurysm height and parent artery diameter is another morphological factor that can be a risk factor of aneurysm rupture. In their study, Detmer et al. found this relationship clearly, but SR was an insignificant factor in ruptured IAs in the study conducted by Liu et al. [19]. Huang et al. discovered that ruptured aneurysms had significantly high SR [21]. Doddasomayajula et al. noted that ruptured IAs were larger in terms of aneurysm volume, aneurysm size, aneurysm area, and SR [25]. Mocco et al. also showed that SR has a significant risk of IA rupture [27]. Consequently, most of the previous studies showed that larger SR, which means larger aneurysms, has a higher risk rate of rupture.

Height, which is defined as the maximum perpendicular distance of the dome from the neck plane, showed no statistical significance in the study conducted by Liu et al. [19]. However, Huang et al. found in their research that the height is significantly related to aneurysm rupture [21]. Doddasomayajula et al. noticed that basilar tip aneurysms possess more risk of rupture than IA bifurcation aneurysms that have a larger height [25]. Mocco et al. noted that perpendicular height is an important predictor for the rupture of IAs [27]. In their study, Skodvin et al. found a statistically significant relationship between height and rupture of an aneurysm [29]. Zhang et al. noticed a significant difference in the maximum height of ruptured aneurysms in comparison with unruptured ones [31]. Accordingly, the majority of these studies linked the height of aneurysm with the risk of rupture, which can, therefore, be used as a predictor while assessing the individual patients.

The bottle-neck factor is defined as the ratio between width, which is the largest diameter that is orthogonal to the greatest distance between the neck plane center and any point on the aneurysm dome, and neck diameter. It is another morphological factor that was studied as a risk for IA rupture. Detmer et al. noticed a significant relationship between the bottle-neck factor and the risk of IA rupture [19]. Another study by Huang et al. also discovered a statistically significant relationship between the bottle-neck factor and ruptured aneurysms [21]. However, this significant relationship could not be seen in the study by Skodvin et al. [29].

A review article conducted by Ambekar et al. found that aneurysms with daughter sacs have more susceptibility to rupture with a hazard ratio of 1.63 [15]. Concurrent to these results, Skodvin et al. noticed in their case series that aneurysms with one or more than one daughter cyst are more liable for rupture [29]. However, no statistically significant difference was found between ruptured and unruptured aneurysms regarding the presence of daughter cysts [19]. Mocco et al. discovered that the presence of the daughter sac is not a statistically significant predictor for aneurysm rupture [27].

Other more complicated parameters such as undulation index, aneurysm (inclination) angle, ellipticity index, non-sphericity index, vessel angle, the relationship among aneurysm neck, parent artery and daughter branches, daughter artery ratio and lateral angle ratio, height-to-width ratio, bulge location, volume-to-ostium ratio, convexity ratio, isoperimetric ratio have been studied too as the risk of rupture in unruptured aneurysms but are still not well established.

Hemodynamic Factors

Computational fluid dynamics: It is a computational technique that makes streamlines (virtual vectors) inside a blood vessel and IA by data analysis from CT or MR angiography or rotational digital subtraction angiography. It is useful for studying fluidodynamics like WSS inside a blood vessel or ICA [32,33].

WSS is the tangential and frictional force exerted by blood flow on the blood vessel wall [23]. It is one of the most studied hemodynamic factors as a risk in the formation, growth, and rupture of IA. Detmer et al. noticed in their study that ruptured IAs have larger maximum wall shear stress (WSSmax), mean WSS (WSSmean), WSS in the aneurysm parent vessel (WSSves), and maximum normalized WSS (MWSSnorm). Interestingly, when normalizing the WSSmean concerning the WSS in the parent artery, it was significantly lower in ruptured aneurysms [17]. In another study, Liu et al. found statistically significant differences between ruptured and unruptured aneurysms regarding the ratio between WSS maximum (WSSm) of aneurysm and WSS maximum (WSSm) of parent vessel (pWSSm). However, there was no significant difference regarding the ratio between WSS average (WSSa) of aneurysm and parent vessel WSS average (pWSSa) [19]. Huang et al. discovered that ruptured aneurysms have larger SAR-TAWSS which is extracted by an equation from the relationship between time-averaged wall shear stress (TAWSS), which is defined as the standard time average of each nodal WSS vector magnitude at the wall across a cardiac cycle [23], and aneurysmal surface area [21]. Qiu et al. noticed that there were significant differences in WSS between the pre-aneurysm surface and near vessel surface before aneurysm, pre-aneurysm surface, and aneurysm surface, and aneurysm surface and near vessel surface after aneurysm [22]. Doddasomayajula et al. noted that ruptured aneurysms had more concentrated WSS distributions and lower minimum WSS (p = 0.003) than unruptured aneurysms [25]. Kaneko et al. found that the average WSS of the aneurysm was 1.2 Pa, which was lower than that of the parent artery 4.2 Pa [26]. The results of the study conducted by Qiu et al. strongly suggest that both high and low WSS were able to cause a rupture in wide-necked aneurysms [28]. In a meta-analysis conducted by Can and Du, there was a positive relationship between high WSS and aneurysm formation and low WSS and aneurysm rupture of bifurcation aneurysms. However, this relationship was negative in sidewall aneurysms [30]. Zhang et al. noted that ruptured aneurysms have lower minimum WSS than unruptured ones [31]. Accordingly, there is a big controversy whether high or low WSS will influence the formation, growth, and rupture of IAs and more sophisticated studies are needed to analyze this issue.

WSS gradient (WSSG) reflects the change in the magnitude of the WSS vector in the flow direction, concerning the streamwise distance [23]. It is used in unruptured IAs with complex geometries and/or with arising vessels [23]. It can be thought of as the change in WSS along the length of the blood vessel [34]. Liu et al. didn't find a statistically significant difference in WSSG between ruptured and unruptured aneurysms [19]. Concomitantly Can and Du in their meta-analysis did not see a significant difference in WSSG between these two groups [30].

Oscillatory shear index (OSI) indicates WSS fluctuations magnitude and describes the tangential force oscillation as a function of the cardiac cycle [23]. OSI measures temporal, rather than spatial variation in the flow direction. Detmer et al. discovered that compared to unruptured aneurysms, ruptured aneurysms had a significantly larger maximum OSI (OSI max) and the mean OSI (OSI mean) [17]. Concomitantly, Liu et al. found a statistically significant difference in WSSG between ruptured and unruptured aneurysms [19]. Huang et al. discovered that ruptured aneurysms have larger SAR-OSI, which is calculated by an equation from the relationship between OSI and aneurysmal surface area [21]. However, in the meta-analysis of Can and Du, the distribution of OSI showed that ruptured aneurysms do not have significantly different pooled OSI compared with unruptured aneurysms [30]. Concomitantly, Zhang et al. found no significant difference in OSI between ruptured and unruptured aneurysms [31].

Low wall shear stress area (LSA) indicates the areas of the aneurysm wall exposed to a WSS value <10% of the mean parent vessel WSS. Liu et al. did not find a statistically significant difference in low wall shear stress area ratio (LSAR normalized by the dome area) between ruptured and unruptured aneurysms [19]. LSA did not reach a statistically significant difference between ruptured and unruptured aneurysms in the observation of Doddasomayajula et al. [25]. On the contrary, Qiu et al. found that LSAR was one of the hemodynamic factors predictive of rupture [22]. The meta-analyses conducted by Can and Du showed significantly higher LSA in the ruptured IAs [30]. Similarly, Zhang et al. also noted that the ruptured aneurysms had significantly more LSA than the unruptured aneurysms [31].

There are many other hemodynamic factors under study like relative residence time, gradient oscillatory number, aneurysm formation indicator, and shear concentration index. Hemodynamic stressors may trigger an inflammatory cascade in the wall of the blood vessels, which may influence the thickness of the blood vessel wall and the morphology and thickness of the wall of IA. Besides, these stressors may induce atherosclerotic changes in the wall of the blood vessel and IA.

Most of the studies were done on ruptured aneurysms and further studies are needed on hemodynamics in unruptured aneurysms to assess the risk of rupture.

Management of unruptured aneurysms: The treatment of unruptured IAs remains challenging. Incorporation of hemodynamic and morphological risk factors after being well studied with well-known risk factors (PHASES score) may yield out a more accurate predictive module for choosing patients with unruptured IAs to be treated by surgical clipping or by endovascular therapy or just observing them.

Limitations: We had to remove a few abstracts for which full text was not retrieved, because of which we may have lost important information. Another limitation was that most of the studies retrieved were observational studies. Therefore, to broaden the horizon and give a better background, we had to include the review articles as well.

## Conclusions

There are well-known risk factors for the rupture of IAs like age, size, and site of the aneurysm. However, the treatment of unruptured IAs remains a significant challenge as this treatment is by invasive procedures and carries high risks. Multiple morphological and hemodynamic parameters of IAs have been studied as risk factors for the rupture of aneurysms. Among geometric factors, AR, SR, aneurysm height, and bottle-neck factor showed significant effects on the growth and rupture of IAs. The reviewed papers also revealed that WSS, OSI, and LSA as hemodynamic factors could have a significant impact on the formation, shape, growth, and rupture of unruptured IAs.

Further studies will be needed to study hemodynamic and morphological factors to yield an accurate predictive module, including well-known risk factors together with hemodynamic and morphological factors, which may be very useful in choosing patients with unruptured IAs for treatment.
